# Use of Antidepressant and Anxiolytic Drugs in Scandinavian Countries between 2006 and 2021: A Prescription Database Study

**DOI:** 10.1155/2024/5448587

**Published:** 2024-01-05

**Authors:** Ivana Bojanić

**Affiliations:** Faculty of Nursing and Health Sciences, Nord University, Levanger, Norway

## Abstract

**Introduction:**

The use of antidepressant and anxiolytic drugs has changed in Scandinavian countries over recent decades, with notable national variations.

**Objective:**

To describe and compare antidepressant and anxiolytic drug use in Norway, Sweden, and Denmark.

**Methods:**

Data included each country's prescription registers from 2006 to 2021. The measures were period (1-year) prevalence (users per 1000 inhabitants) and therapeutic intensity (TI; daily defined dose (DDD) per 1000 inhabitants per day), overall, by drug classes and age groups.

**Results:**

The prevalence of antidepressant use increased from 2006 to 2021 and was highest in Sweden (78 to 107 users per 1000 inhabitants) and lowest in Norway (61 to 69 users per 1000 inhabitants). The prevalence of anxiolytic use decreased, most steeply in Denmark (50 to 18 users per 1000 inhabitants). The TI of antidepressants increased consistently in Norway and Sweden, but more variably in Denmark. Sweden had the highest increase in TI of antidepressants (56%). The TI of anxiolytics declined most markedly in Denmark (by 75%). The prevalence of antidepressant and anxiolytic use was highest among adults ≥65 years. The prevalence of antidepressant use increased across age groups in Sweden and young people (5-19 years) in Norway, but not in Denmark.

**Conclusions:**

The use of antidepressants increased in Scandinavia in 2006-2021, but decreased for anxiolytics, with country variations in the number of users and the amount used. Future research should target factors underlying high antidepressant and anxiolytic use in older adults across countries and increasing antidepressant use in Sweden and among young Norwegians.

## 1. Introduction

Globally, it is estimated that over 700 million people suffer from mental health disorders [1], putting them among the leading causes of health and economic losses [2]. Antidepressants and anxiolytics are psychotropic drugs, effective and often recommended as first-line treatment of various mental health disorders [3]. In recent decades, there has been a global rise in antidepressant and anxiolytic use, particularly in highly developed countries [4]. This trend may be attributed to the increasing prevalence of relevant mental health disorders [5], expanded drug indications [6], and the introduction of generic alternatives to the market [7]. A comprehensive study across more than 65 regions worldwide revealed significant variations in the consumption of psychotropic drugs between 2008 and 2019, with the highest use in high- and middle-high-income countries [4]. Among European countries outside Scandinavia, the study reported the highest use of antidepressants in the United Kingdom (UK), Portugal, Ireland, and Spain, ranging from 87 to 124 daily-defined doses per 1000 inhabitants per day. Despite the geographical proximity and similar income levels, the same study showed a significant variation in drug use across countries. Notably, the UK has seen higher antidepressant use than Germany and France (124 vs. 11 daily defined doses per 1000 inhabitants per day), while both the UK and Germany had similar anxiolytic use, both lower than France (11 vs. 29 daily defined doses per 1000 inhabitants per day) in 2019 [4]. Thus, the observed disparities in antidepressant and anxiolytic drug use among countries can only be partially explained by geographical location and income [4]. Other socioeconomic and demographic factors affecting drug use trends may include, but are not restricted to, the country-level burdens of mental illness [4, 5], life expectancy [2], and health expenditure [2, 4].

In 2020, Scandinavian countries ranked among the top ten users of antidepressants in Europe while exhibiting lower anxiolytic use compared to other countries [8]. Despite their comparable culture, economies [9], and prevalence of common mental health disorders (i.e., depression and anxiety) [10], recent studies suggest differences both in overall use and age-specific use of antidepressants and anxiolytics between Scandinavian countries over the last two decades [11–13]. Notably, the use of anxiolytics reduced overall in Scandinavia from 2004 to 2020, but the decrease was most marked in Denmark, compared to Norway and Sweden [11]. A population-based study including nearly one million individuals aged 5-19 years reported an over twofold increase in the prevalence of antidepressant users (from 9.3 to 18.0 users per 1000 inhabitants) in Sweden and a smaller rise in Norway, while there was a decrease in Denmark from 2007 to 2017 [12]. The observed discrepancies in antidepressant and anxiolytic use may be attributed to various factors, such as public attitudes and awareness about mental health disorders [14], availability of mental health treatment options [5], and prescribing practices [10] that may differ between countries. There are growing concerns about the appropriate use of these drugs in relation to long-term treatment [15], the potential for overprescribing [16], and their safety among paediatric [17, 18] and elderly users [19, 20].

The available register data [21] and compatibility between Norway, Sweden, and Denmark [10] allow us to examine and compare trends in antidepressant and anxiolytic use across countries and over time. Earlier studies have been limited to specific populations [12, 15, 22], one drug class [11, 15], one country [15, 23, 24], or had shorter study periods [15, 23]. To inform future initiatives on drug prescription, revision of clinical guidelines, or allocation of economic resources, investigations of variation in drug use in general populations are needed. This study investigates the use of antidepressant and anxiolytic drugs in Scandinavian countries between 2006 and 2021 in order to describe and compare their use between the countries, overall, by drug classes and across age groups.

## 2. Methods

### 2.1. Data Sources

Data material was collected from the national prescription registers in Norway [25], Sweden [26], and Denmark [27]. These national prescription registers include dispensed drug prescriptions from community pharmacies (i.e., expressed in the number of users and doses) by drug classes and age groups. 98-99% of prescriptions can be identified individually, thus providing valid estimates of drug use [21]. As data on the total amount of drugs dispensed are not available from the Swedish Prescription Drug Register, the Nordic Medico-Statistical Committees (NOMESCO) database was used as a supplementary data source. NOMESCO data are described elsewhere (see https://nhwstat.org/health/pharmaceutical-products). See Table 1 for more information on the data sources.

### 2.2. Antidepressant and Anxiolytic Drugs

Drug classes within the Anatomical Therapeutic Chemical (ATC) group N06A (antidepressants) and N05B (anxiolytics) were analyzed. Antidepressants included N06AA (nonselective monoamine reuptake inhibitors, tricyclic antidepressants, TCAs), N06AB (selective serotonin reuptake inhibitors, SSRIs), and N06AX (defined as “other antidepressants”). “Other antidepressants” further classified into specific drug classes (ATC 5^th^ level) predominantly used in the countries are included in Supplementary Table S3. Anxiolytics included N05BA (benzodiazepine derivatives, BZDs), N05BB (diphenylmethane derivatives), and N05BE (azaspirodecanedione derivatives).

### 2.3. Main Measures

To investigate drug use in terms of the number of users and the amount used, the following measurements were calculated: (i) period (one-year) prevalence as the number of users per 1000 inhabitants per year and (ii) therapeutic intensity (TI) as the number of WHO-defined daily doses (DDD) used per 1000 inhabitants per day. DDD is a standardized measure of drug amount used based on the “assumed average maintenance dose per day for a drug used for its main indication in adults” [28].

### 2.4. Statistical Analysis

The analysis included all available antidepressant and anxiolytic drugs in the Scandinavian countries, overall and by each drug class. Prevalence was analyzed overall and by the following age groups: 5-19, 20-64, and ≥65 years. Supplementary analysis, overall and by sexes, included other age groups: 5-14, 15-19, 20-44, 45-64, 65-74, and ≥75 years. Sex stratification for the age group 5-14 years was excluded due to a low number of users per 1000 inhabitants. For further information on supplementary analysis, see section description of supplementary material. Prevalence of drug use was calculated as the number of individuals with one or more drug prescriptions within a calendar year divided by the number of inhabitants in thousands in that year (yielding users per 1000 inhabitants). TI was calculated as the total number of DDDs used per calendar year divided by the total number of inhabitants in the same year and divided by 365 days per year (yielding DDD per 1000 inhabitants per day).

The calculations were based on data from prescription registers and available national statistics. For Sweden, data on drug use in DDD per 1000 inhabitants per day were only available for anxiolytics overall and for the subgroup BZDs from NOMESCO. Therefore, prescriptions per 1000 inhabitants per year collected from the National Swedish Drug Registry were used in the calculations. DDD per 1000 inhabitants per day was calculated as the product of the number of prescriptions, drug mass, and average dispensed quantity multiplied by 1000 and divided by DDD, population, and days in the year [29]. The TI and total dose (as 1000 DDD) were calculated for two specific ATC level 5 approved drug groups in Sweden: diphenylmet. derivatives (N05BB01) and azasp. derivatives (N05BE).

The changes in one-year prevalence and TI over time were calculated as relative changes in percentage. Trend analysis included a test for trend with associated *p* values for the hypothesis of no trend. The results in tables and figures display data at three-year intervals for ease of presentation. However, the statistical trend analysis utilizes data from each year. All calculations were done with STATA release 17 (StataCorp, College Station, TX, USA).

### 2.5. Ethics

This descriptive drug utilization study used routinely collected (deidentified) data from publicly available sources for which no informed consent or ethical approval is necessary.

## 3. Results

### 3.1. Trends in Antidepressant and Anxiolytic Drug Use

**Figure 1:**
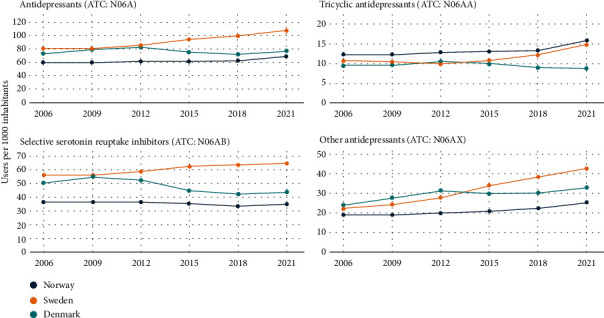
Time trend in the prevalence of antidepressant drug use (number of users per 1000 inhabitants) by drug class and country from 2006 to 2021. Note: variation in *y*-axes. ATC: Anatomical Therapeutic Chemical Classification.

**Figure 2:**
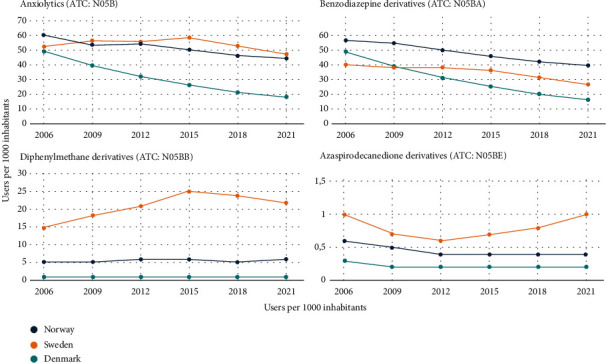
Time trend in the prevalence of anxiolytic drug use (number of users per 1000 inhabitants) by drug class and country from 2006 to 2021. Note: variation in *y*-axes. ATC: Anatomical Therapeutic Chemical Classification.

The overall use of antidepressant and anxiolytic drug groups was presented in the total number of users, one-year prevalence (users per 1000 inhabitants), and relative changes in prevalence (Tables 2 and 3). Figures 1 and 2 show the time trend in the prevalence of antidepressant and anxiolytic drug use (number of users per 1000 inhabitants) by drug class and country from 2006 to 2021.

During the study period, the use of antidepressants increased steadily across Scandinavia (Figure 1). The prevalence of antidepressant use was consistently highest in Sweden (from 78 to 107 users per 1000 inhabitants), followed by Denmark, and lowest in Norway (from 60 to 69 users per 1000 inhabitants) (Figure 1). However, in terms of relative change in the prevalence of antidepressant use, Sweden had the greatest increase by 34%, while the lowest increase was in Denmark by 5% (Table 2). SSRIs had the highest prevalence throughout the study but with notable variations between the countries. The prevalence of SSRI use increased by 15% in Sweden, reduced by nearly 13% in Denmark, and remained relatively stable in Norway (Table 2 and Figure 1). However, the prevalence of “other antidepressant” use increased steadily across all three countries; the relative increase in Sweden was nearly three times greater than in Norway or Denmark. Conversely, the prevalence of TCA use increased in Sweden and Norway but decreased in Denmark. Among group “other antidepressants,” there has been a notable increase in the use of serotonin-norepinephrine reuptake inhibitors, SNRIs (i.e., duloxetine and venlafaxine) and atypical antidepressant mirtazapine across countries (Table S3). This rise has coincided with a concurrent decrease in the utilization of tetracyclic antidepressants (i.e., mianserin).

The prevalence of anxiolytic use declined consistently across Scandinavia (Figure 2); however, the largest decline was in Denmark (64%), followed by Norway (26%) and Sweden (10%). BZDs accounted for the most anxiolytic use in all three countries. However, the prevalence of diphenylmet. derivatives increased markedly, with Sweden showing the highest relative increase (47%, from 15 to 22 users per 1000 inhabitants) compared to Norway and Denmark (Table 3).

Overall, the TI of antidepressants increased across all three countries, but with notable differences in levels of TI and relative change throughout the period (Table 4). In Norway and Sweden, the TI of antidepressants rose consistently, while in Denmark, it increased initially (from 2006 to 2012), followed by a decline to 2021. However, the largest relative change in TI was in Sweden with a 56% increase, followed by Denmark (31%) and Norway (13%). Sweden had the highest TI of antidepressants in both 2006 and 2021 (from 69.7 and 108.9 DDD per 1000 inhabitants per day, respectively), while the levels of TI of antidepressants were consistently lowest in Norway. TI of SSRI use increased in all countries, with the largest relative increase in Sweden (41%) and the lowest in Norway (6%). Notably, there was a rising trend in TI of “other antidepressants” across the three countries, most markedly in Sweden (119%), followed by Denmark (81%) and Norway (36%). TI of anxiolytics decreased in all three countries, mainly attributed to reduced BZD use; this trend was most marked in Denmark (75%) then Norway (44%) and Sweden (39%) (Table 5).

### 3.2. The Antidepressant and Anxiolytic Drug Use across Age Groups

**Figure 3:**
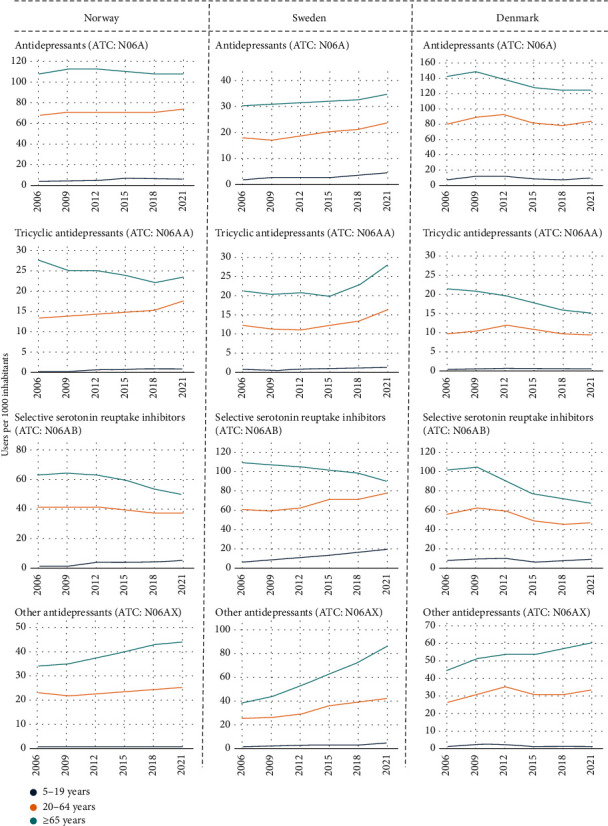
Time trend in the prevalence of antidepressant drug use (number of users per 1000 inhabitants) by age groups, drug class, and country. Note: *y*-axis differs between countries. ATC: Anatomical Therapeutic Chemical Classification.

**Figure 4:**
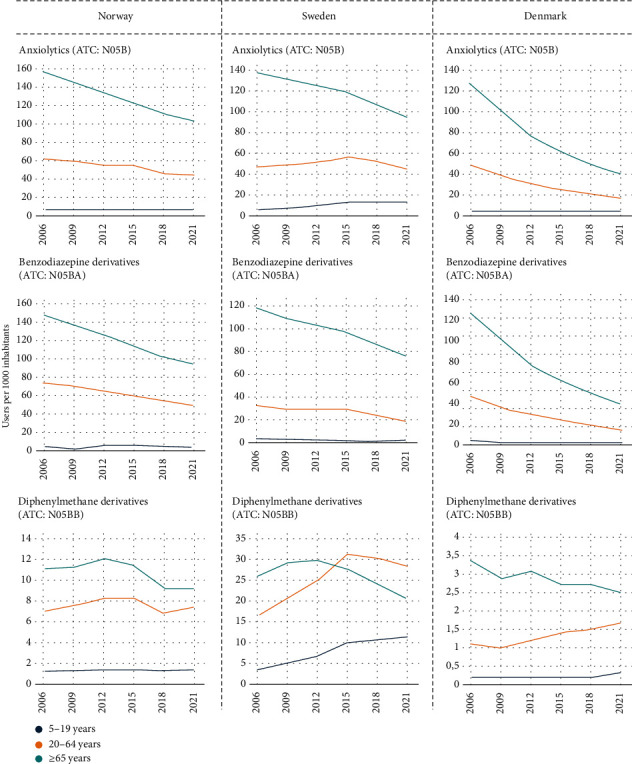
Time trend in the prevalence of anxiolytic drug use (number of users per 1000 inhabitants) by age groups, drug class, and country. Note: *y*-axis differs between countries. ATC: Anatomical Therapeutic Chemical Classification.

The use of antidepressants and anxiolytic drugs increased with age across the three countries, with the highest prevalence observed in adults ≥65 years (Figures 3 and 4). In contrast to Norway and Denmark, there was a rising trend in the prevalence of antidepressant use across all age groups in Sweden from 2006 to 2021. This was sharpest in individuals aged 5-19 years (204%; from 8 to 24 users per 1000 inhabitants) and lowest among those ≥65 years (16%). A similar trend was observed in Norway in the group aged 5-19 years but not in Denmark. Notably, the prevalence of antidepressant use remained relatively stable in Norway but declined in Denmark in those ≥65 years (Figure 3 and Table S1).

SSRIs dominated antidepressant use across age groups in all three countries. However, there was a decrease in the prevalence of SSRI use among individuals ≥65 years, most markedly in Denmark (by 33%; from 101 to 68 users per 1000 inhabitants), but this was accompanied by an increase in the prevalence of “other antidepressant” use. The prevalence of antidepressants used showed very small differences between the age groups 45-65 and 65-75 years; however, the highest was those ≥75 years throughout the period in all three countries (Figure S1).

The prevalence of anxiolytic use declined across all age groups in Denmark and Norway, but this trend was only observed in the age group ≥65 years in Sweden (Figure 4 and Table S2). Notably, anxiolytic use increased markedly among Swedish individuals aged 5-19 years, and this contributed to the increasing use of diphenylmeth derivatives. Age differences in the prevalence of anxiolytic drug use, mainly due to BZDs, were the largest in Sweden and were minimal in Denmark. However, the prevalence of BZD use consistently remained highest in adults aged ≥75 years in all three countries throughout the study period (Figure S2).

Throughout the study period, women consistently exhibited approximately two times higher prevalence of overall antidepressant and anxiolytic use compared to men across all age groups and countries (Supplementary analysis Tables S4–S7). Overall, younger individuals (15-19 years and 20-44 years) experienced a higher relative increase in total antidepressant use compared to older ones for both sexes (Table S4 and S5). Swedish women aged 15-19 years and 20-44 years consistently showed the highest prevalence of antidepressant use compared to their counterparts in Denmark and Norway. In 2021, young women aged 15-19 years in Sweden showed an antidepressant use prevalence almost three times higher than their counterparts of the same age in Norway and Denmark. In contrast to their Norwegian and Danish peers, there has been an increase in the prevalence of overall anxiolytic use among young women and men (aged 15-19 years and 20-44 years) in Sweden from 2006 to 2021 (Table S6 and S7).

## 4. Discussion

This study showed that overall antidepressant use in Scandinavia between 2006 and 2021 increased, but anxiolytic use decreased; however, there were notable differences between the countries and between age groups. Sweden consistently had the highest use of antidepressant drugs in Scandinavia, while the relative decline in anxiolytic drug use was highest in Denmark. Overall, antidepressant and anxiolytic drug use increased with increasing age and was highest in older age groups (≥75 years). Antidepressant use showed increases across all age groups in Sweden and particularly in individuals aged 15-19 years in Norway during the study period, but no such trend was observed in Denmark. There was a marked increase in anxiolytic drug use in individuals aged 15-19 years in Sweden, in contrast to their Danish and Norwegian peers. Across all age groups and countries, women consistently exhibited a higher prevalence of overall antidepressant and anxiolytic use compared to men, the trend notably pronounced among young Swedish women.

As antidepressants are the first-line treatment for depression and anxiety disorders [6], it is likely that these disorders are driving the increasing use of antidepressants in Scandinavian and other European countries [4]. Hence, the growing trend of antidepressant use in Norway, Sweden, and Denmark may be partly attributed to the increased prevalence of current mental health disorders (i.e., depression and anxiety) in Scandinavia [10]. This, in turn, may reflect the global mental health burden in the developed countries [30, 31]. The availability of new-generation antidepressants with improved risk-benefit profiles and expansion of their indications to other psychiatric disorders (insomnia, manic-depressive disorders, eating disorders, etc.) and physical conditions (e.g., peripheral neuropathic pain, migraine, etc.) [6] has probably contributed to increased antidepressant use. The changes in guidelines for treating anxiety, which recommend antidepressants as a first-line treatment during the study period (see Table 6), could be partly responsible for the increasing antidepressant use observed in all three studied countries. Moreover, the emergence of “other antidepressants” (ATC: N06AX) with diverse mechanisms of action provides an alternative treatment avenue, particularly for patients resistant to standard therapies like SSRIs. In line with this study's findings (Table S3), there has been a noticeable shift towards using SNRIs (i.e., duloxetine and venlafaxine) to treat major depressive disorders in Scandinavian countries, following the use of SSRIs [10]. In line with our findings, newer classes of antidepressants, including SSRIs, SNRIs, and other antidepressants, are preferred over older TCA globally, although there is a growing trend in lower-middle-income countries favouring older, potentially more affordable antidepressants [4]. However, atypical tetracyclic antidepressants like mirtazapine are prescribed for conditions beyond depression or anxiety, such as chronic pain and sleep disorders. This broader usage might contribute to the rise in their use in Scandinavia, as indicated by the findings of this study.

Scandinavian countries share strong cultural, economic, and health service similarities (e.g., free access to healthcare and funding for medication) [9, 32]. All three countries also have increased public awareness of and decreasing social stigma towards mental health disorders [33]. These factors collectively may have led to more individuals seeking help and receiving antidepressants over the past few years and contributed to the increased use of antidepressants in these countries. On the other hand, the results of this study showed a markedly higher overall use of antidepressants in Sweden throughout the period (2006-2021). A cross-country comparison between Baltic and Nordic countries reported a similar trend in antidepressant use in the Baltic region (containing the countries Estonia, Latvia, and Lithuania) in 2010-2015, while Sweden ranked highest in antidepressant use compared to Norway, Denmark, and Baltic countries in 2015, albeit below Iceland and Finland [13].

The reasons for the varying antidepressant use in Scandinavian countries remain unknown. There is a comparable prevalence of psychiatric disease diagnosis, and there are similar treatment guidelines for depression and anxiety disorders in these countries. A recent prescription database study showed a much higher prevalence of common psychiatric disorders (e.g., anxiety and depression) and certain clinical outcomes (e.g., major depressive disorders and generalized anxiety disorders) in Sweden compared to Norway and Denmark [10]. There was also a higher frequency of mental health visits to and antidepressant prescriptions made in specialized rather than primary healthcare compared to Norway and Denmark [10]. Access to healthcare in Norway and Denmark is largely centralized through general practitioners (GPs), whereas patients in Sweden often seek direct access to specialist services and psychiatrists [32]. All these variations in prevalence, access to services, and treatment approaches (including diagnosis and healthcare pathways) in Scandinavia may contribute to differences in antidepressant use between the countries.

The overall reduction in anxiolytic drug use, mainly driven by BZD use, may reflect the several recommendations and regulations initiated in Scandinavian countries before and during the study period (see Table 6). These were aligned with international recommendations and perceptions of BZD-related risks [34]. However, this alone does not explain the notably higher drop in anxiolytic use, mainly BZD, in Denmark. There was a large number of local and national public health initiatives addressing BZDs in Denmark from 2003 to 2013 [35]. These could have resulted in a greater reduction in their use compared to the other two countries. Despite documented adverse events in the older population, including increased risk of falls [19] and cognitive impairment [36], BZD use still remains high in these age groups in Europe [11], Canada [37], and the USA [38]. A recent drug use study showed that the highest BZD use was in older age groups (≥80 years) in all Nordic countries [11], and the results are in agreement with the findings in this study. Unfortunately, the drivers of these age-specific patterns in BZD use are still unclear; they could be due to the increasing number of new users and/or insufficient deprescribing for long-term users in the older population [39].

Furthermore, previous studies have also highlighted the increasing trend in overall antidepressant and anxiolytic use [22] and BZD use in younger age groups (i.e., children and adolescents (0-17 years) and young adults (18-24 years)) in Sweden [15], specifically among females, the findings supported by this study. A Swedish population-based register study showed that the substantial rise in the prevalence of BZD use in younger age groups between 2006 and 2013 was followed by high prevalence of off-label and long-term (>6 months) use, psychotropic drug polypharmacy and prescriptions from primary care or nonpsychiatric institutions, which may indicate nonadherence to BZD prescribing guidelines [15]. However, when we interpret the results of BZD use in younger age groups, it is important to remember that there is no firmly established indication for BZD treatment in children and adolescents in psychiatry [17]—and that is also true in Sweden.

Unlike prior studies restricted by short timeframes [15], singular drug class analyses [11, 12], age group [16–18], or focusing on a single country [16], this study is a leap forward. Examining both antidepressants and anxiolytics across countries, ages, and sexes in a unified framework offers a more comprehensive picture of trends in the use of the primary medications for treating depression and anxiety in Scandinavia, where these conditions pose a substantial burden of disease. These results may provide thorough information on similarities and differences in the use of these drugs across Scandinavia that may have been overlooked in isolated or fragmented analyses. Other strengths of this study include the use of three nationwide population databases with validated and nearly complete data on drug use in the entire population. There is minimal (no) selection and recall bias or loss of follow-up. However, there are some limitations in the use of these data sources which include a lack of information on indications and duration of drug treatment and whether the decline in drug use was due to changing to other drug classes (e.g., from BZDs to antidepressants, antihistamines, or low dose antipsychotics) or change in therapy. The data utilized in this study are aggregated and deidentified, meaning that prescriptions of studied drugs cannot be linked to specific individuals, making it not possible to distinguish between those using a single drug from those using several drugs (i.e., polypharmacy). Consequently, drug subgroups are not mutually exclusive. The prevalence of drug use did not include drugs dispensed to patients in hospitals or secondary health institutions. This could lead to underestimation without necessarily affecting the observed trends. Moreover, this study could not differentiate new users (i.e., incident users) from individuals already using antidepressants or anxiolytics, or those using several antidepressants or anxiolytics (i.e., engaging in polypharmacy), so overestimation of the point prevalence for the use of each drug is likely. Importantly, DDD is not the clinically recommended therapeutic dose but is the daily maintenance dose used for the drug's main indication in adults; hence, different doses may be recommended for different conditions. For example, TCA amitriptyline is used in higher doses for depression (main indication) than in neuropathic pain or migraine, which may overestimate TI for this drug class. Therefore, the TI of antidepressant and anxiolytic use should be interpreted as suggestive. Furthermore, antidepressant and anxiolytic drug use in the age group 5-14 years may be underestimated because of the lack of paediatric drug formulations within these drug classes. Finally, the absence of other factors (e.g., education, cohabitation status, income, alcohol intake, and health conditions) that impact drug use at both individual and population levels [40–42] is an important limitation in this study that needs to be considered in the interpretation of the findings.

In conclusion, antidepressant use increased while anxiolytic use decreased between 2006 and 2021 in Scandinavia, but with differences by country and age group. The high prevalence of antidepressant and anxiolytic use among older adults (≥75 years) and the rising trend in their use among Swedish young people (15-19 years) are urgent reasons for research and public health interventions. These findings highlight the need for drug use studies that include socioeconomic, demographic, and health-related characteristics as important determinants of drug use on individual and population levels to inform public health initiatives and development of better guidelines. Moreover, future drug utilization studies need to thoroughly explore social, economic, and geographical (e.g., rural vs. urban) characteristics within each country's basic health zones to fully understand the factors behind increased drug use, moving beyond mere country-level analysis.

## Figures and Tables

**Table 1:** Description of registers, coverage, and obtained data.

Country	Data source	Coverage (period and use)
Denmark	The Danish Register of Medicinal Product Statistics, the Danish Health Data Authority (http://www.medstat.dk)	2000-2022 (individual use and wholesale data) Includes all prescriptions filled at community pharmacies

Norway	The Norwegian Prescription Database, the Norwegian Institute of Public Health (http://www.norpd.no)	2004-2020 (individual use (users and amounts))

Sweden	The Swedish Prescribed Drug Register, Socialstyrelsen (http://www.socialstyrelsen.se)	2006-2022 (individual use (users))

All Nordic countries	Nordic Medico-Statistical Committees database (NOMESCO) (http://www.nhwstat.org)	2006-2021 (individual use (users and amounts)) Includes all prescriptions filled at the primary and hospital sector

**Table 2:** Total number of users (users; no) and prevalence (prev.; users per 1000 inhabitants) of antidepressants by drug class and country from 2006 to 2021.

Drug group	2006		2009		2012		2015		2018		2021		Relative change (%)^a^	*P* ^b^
	Users	Prev.	Users	Prev.	Users	Prev.	Users	Prev.	Users	Prev.	Users	Prev.		
*Antidepressants (N06A)*														
Norway	279 501	60.0	292 389	60.6	309 879	61.8	322 638	62.2	332 866	62.7	371 439	68.9	+14.8	
Sweden	720 881	79.7	739 748	79.9	808 510	85.3	918 713	94.2	1 004 421	99.2	1 109 540	106.9	+34.1	
Denmark	394 980	72.8	441 805	80.2	458 490	82.2	420 890	74.4	417 645	72.2	447 415	76.6	+5.2	≥0.05

*TCA (N06AA)*														
Norway	57 551	12.3	60 235	12.5	64 718	12.9	68 306	13.2	71 160	13.4	85 569	15.9	+29.3	
Sweden	98 299	10.9	93 677	10.1	98 444	10.4	105 989	10.9	123 845	12.2	155 368	15.0	+37.6	≥0.05
Denmark	51 100	9.4	54 355	9.9	59 350	10.6	55 855	9.9	51 965	9.0	50 690	8.7	-7.4	

*SSRI (N06AB)*														
Norway	169 289	36.3	178 925	37.0	186 235	37.1	185 844	35.8	179 802	33.8	191 145	35.5	-2.2	≥0.05
Sweden	509 963	56.4	517 135	55.9	558 075	58.8	616 488	63.2	639 910	63.2	674 917	65.0	+15.2	
Denmark	273 530	50.4	304 060	55.2	293 190	52.5	254 205	44.9	242 975	42.0	257 240	44.0	-12.5	≥0.05

*Other antidepressants (N06AX)*														
Norway	88 876	19.1	90 568	18.8	98 709	19.7	107 668	20.7	118 284	22.3	134 022	24.9	+30.4	
Sweden	200 552	22.2	222 811	24.1	263 207	27.8	332 087	34.1	387 789	38.3	443 278	42.7	+92.3	
Denmark	128 220	23.6	150 715	27.3	174 310	31.2	166 575	29.4	173 495	30.0	191 020	32.7	+36.4	≥0.05

Abbreviations: TCA: tricyclic antidepressants; SSRI: selective serotonin reuptake inhibitors. The table displays data points every three years for ease of presentation, but the statistical trend analysis and graphs are based on data from each year. ^a^Percentage differences in the year 2021 compared to the year 2006. ^b^Jonckheere-Terpstra trend test *P* value for trend analysis ≥ 0.05.

**Table 3:** Total number of users (users; no) and prevalence (prev.; users per 1000 inhabitants) of anxiolytics by drug class and country from 2006 to 2021.

Drug group	2006		2009		2012		2015		2018		2021		Relative change (%)^a^	*P* ^b^
	Users	Prev.	Users	Prev.	Users	Prev.	Users	Prev.	Users	Prev.	Users	Prev.		
*Anxiolytics (N05B)*														
Norway	281 237	60.3	282 061	58.4	273 786	54.5	262 410	50.6	246 975	46.5	239 720	44.5	-26.2	
Sweden	475 176	52.5	496 196	53.6	526 926	55.6	569 184	58.4	540 223	53.4	489 933	47.2	-10.1	≥0.05
Denmark	271 820	50.1	217 730	39.5	180 030	32.3	150 730	26.6	125 280	21.7	105 050	18.0	-64.1	

*BZDs (N05BA)*														
Norway	261 614	56.1	261 065	54.1	249 510	49.7	236 178	45.5	223 508	42.1	211 583	39.2	-30.1	
Sweden	362 499	40.1	355 574	38.4	359 546	37.9	356 040	36.5	322 088	31.8	275 274	26.5	-33.9	
Denmark	265 880	49.0	212 115	38.5	173 315	31.1	143 630	25.4	117 760	20.4	96 615	16.5	-66.3	

*Diphenylmet. derivatives (N05BB)*														
Norway	25 710	5.5	28 280	5.8	32 309	6.4	34 013	6.5	29 878	5.6	34 391	6.4	+16.4	≥0.05
Sweden	137 428	15.2	173 137	18.7	203 218	21.4	249 524	25.6	244 556	24.2	231 336	22.3	+46.7	≥0.05
Denmark	6 670	1.2	6 005	1.1	7 210	1.3	7 750	1.4	8 160	1.4	8 875	1.5	+25.0	

*Azasp. derivatives (N05BE)*														
Norway	2 965	0.6	2 394	0.5	2 576	0.5	2 270	0.4	2 051	0.4	2 201	0.4	-33.3	
Sweden	9 475	1.0	6 918	0.7	5 840	0.6	6 622	0.7	8 039	0.8	10 277	1.0	—	
Denmark	1 400	0.3	1 420	0.3	1 160	0.2	1 065	0.2	965	0.2	1 090	0.2	-33.3	≥0.05

Abbreviations: BZDs: benzodiazepine derivatives; diphenylmet. derivatives: diphenylmethane derivatives; azasp. derivatives: azaspirodecanedione derivatives. The table displays data points every three years for ease of presentation, but the statistical trend analysis and graphs are based on data from each year. ^a^Percentage differences in the year 2021 compared to the year 2006. ^b^Jonckheere-Terpstra trend test *P* value for trend *analysis* ≥ 0.05.

**Table 4:** Time trends in therapeutic intensity (TI; DDD per 1000 inhabitants per day) and relative change in TI (in %) of antidepressants by drug class and country from 2006 to 2021.

Drug group	DDD/1000 inhabitants/day						Relative change (%)^a^	*P* ^b^
	2006	2009	2012	2015	2018	2021⁣^∗^		
*Antidepressants (N06A)*								
Norway	49.0	51.6	53.2	53.5	53.0	55.4	+13.1	
Sweden⁣^∗∗^	69.7	74.1	81.1	92.5	98.7	108.9	+56.2	
Denmark	63.8	77.1	82.1	76.0	75.7	83.8	+31.3	≥0.05

*TCA (N06AA)*								
Norway	3.6	3.5	3.4	3.4	3.3	3.6	—	
Sweden⁣^∗∗^	3.8	3.5	3.4	3.4	3.6	4.1	+7.9	≥0.05
Denmark	4.2	4.4	4.7	4.3	3.8	3.6	-14.3	≥0.05

*SSRI (N06AB)*								
Norway	32.6	34.6	35.5	35.1	33.8	34.7	+6.4	≥0.05
Sweden⁣^∗∗^	50.5	52.4	56.0	62.5	65.1	71.4	+41.4	
Denmark	44.1	52.8	52.4	47.1	46.7	52.5	+19.0	≥0.05

*Other antidepressants (N06AX)*								
Norway	12.5	13.3	14.1	14.9	15.9	17.1	+36.8	
Sweden⁣^∗∗^	15.2	18.0	21.5	26.6	29.9	33.3	+119.1	
Denmark	15.2	19.8	24.9	24.5	25.1	27.6	+81.6	

Abbreviations: DDD: defined daily doses; TCA: tricyclic antidepressants; SSRI: selective serotonin reuptake inhibitors. The table displays data points every three years for ease of presentation, but the statistical trend analysis and graphs are based on data from each year. Notes: ⁣^∗^Data in the 2021 column and relative change (%) for Norway were calculated for 2020 as this was the last year with publicly available prescription data. Data in the 2021 column and relative change (%) for remaining countries were calculated for 2021.⁣^∗∗^Data from Sweden include both sales at community pharmacies and hospital use, while data from remaining countries cover only sales at community pharmacies. ^a^Percentage differences in the year 2021 compared to the year 2006. ^b^Jonckheere-Terpstra trend test *P* value for trend a*nalysis* ≥ 0.05.

**Table 5:** Time trends in therapeutic intensity (TI; DDD per 1000 inhabitants per day) and relative change in TI (%) of anxiolytics by drug class and country from 2006 to 2021.

Drug group	DDD/1000 inhabitants/day						Relative change (%)^a^	*P* ^b^
	2006	2009	2012	2015	2018	2021⁣^∗^		
*Anxiolytics (N05B)*								
Norway	19.2	18.9	15.8	13.4	11.1	10.7	-44.3	
Sweden⁣^∗∗^	16.4	16.2	15.5	14.5	11.9	10.0	-39.0	
Denmark	17.9	12.6	9.8	7.8	5.6	4.4	-75.4	

*BZDs (N05BA)*								
Norway	18.2	17.1	14.5	12.1	10.1	9.7	-46.7	
Sweden⁣^∗∗^	13.5	12.9	12.1	10.6	8.3	6.5	-51.9	
Denmark	17.7	12.3	9.5	7.5	5.2	4.0	-77.4	

*Diphenlymet. derivatives (N05BB)*								
Norway	0.7	1.0	1.0	1.1	0.9	0.9	+28.6	≥0.05
Sweden⁣^∗∗^	2.0	2.4	2.5	2.7	2.5	2.4	+20.0	≥0.05
Denmark	0.2	0.2	0.2	0.3	0.3	0.3	+50.0	≥0.05

*Azasp. derivatives (N05BE)*								
Norway	0.3	0.2	0.2	0.2	0.1	0.2	-33.3	≥0.05
Sweden⁣^∗∗^	0.8	0.6	0.5	0.6	0.6	0.8	—	
Denmark	0.1	0.1	0.1	0.1	0.1	0.1	—	

Abbreviations: BZDs: benzodiazepine derivatives; diphenylmet. derivatives: diphenylmethane derivatives; azasp. derivatives: azaspirodecanedione derivatives. The table displays data points every three years for ease of presentation, but the statistical trend analysis and graphs are based on data from each year. Notes: ⁣^∗^Data in the 2021 column and relative change (%) for Norway were calculated for 2020 as this was the last year with publicly available prescription data. Data in the 2021 column and relative change (%) for remaining countries were calculated for 2021. ⁣^∗∗^Data from Sweden include both sales at community pharmacies and hospital use, while data from remaining countries cover only sales at community pharmacies. ^a^Percentage differences in the year 2021 compared to the year 2006. ^b^Jonckheere-Terpstra trend test *P* value for trend *analysis* ≥ 0.05.

**Table 6:** Timeline of specific recommendations or regulations in Scandinavian countries on the use of psychotropic drugs.

Country	Year	Recommendations or regulations
Norway	1990	Guide to the prescription of addictive drugs
	2001	Revised guide on addictive drugs. Prescribing and justification, only in electronic format
	2003	Restriction in the prescription status from group B (addictive) to A (highly addictive) for flunitrazepam
	2014	Guide to the prescription of addictive drugs
	2021	The guide replaces the following guides: National Professional Guide for addictive drugs 2014—requisition and professional soundness and national professional guide for the use of opioids for long-term noncancer-related pain. The main lines of the professional recommendations for symptom-relieving treatment with benzodiazepines, benzodiazepine-like drugs, and opioids are continued. The new guide is concise and more user-friendly than previous versions. The guide gives a reminder to treating physicians that there is room for quality improvements both in treatment choices and in follow-up of patients with especially anxiety, sleep problems, or long-term pain conditions that are not due to cancer.
Sweden	2004	National Indicators for Quality of Drug Therapy in Older Persons (National Board of Health and Welfare). Long-acting benzodiazepines should be avoided in persons aged ≥70 years.
	2006	Treatment recommendations for anxiety (Swedish Medical Products Agency). Benzodiazepines to be avoided in anxiety disorders (Swedish Medical Products Agency)
	2009-2010	National guidelines for treatment for depression and anxiety (National Board of Health and Welfare). Recommends benzodiazepines as the last treatment option and should only be used as short-term treatment.
	2017	New national guidelines for treatment for depression and anxiety (National Board of Health and Welfare). Benzodiazepines should not be used to treat GAD, anxiety disorders, PTSD, etc.
Denmark	1980	The first guideline on the prescription of drugs drug causing dependency (benzodiazepines, opioids, and psychostimulants)
	2003	Ministerial initiatives on surveillance of benzodiazepine and benzodiazepine-related drugs
	2008	Guidelines on the prescription of drugs causing dependency—updated. Focus on benzodiazepines and driving
	2010	Guideline on anxiety disorders for general practitioners—treatment with benzodiazepines should be not a first-line treatment and preferably for short-term treatment.
	2014	Guideline on treatment with antipsychotic drugs to individuals older than 18 years with psychotic disorders. Treatment with benzodiazepines for agitation should be of short duration. A combination of antipsychotics and benzodiazepines should be avoided due to the increased risk of death.
	2017	Electronic prescriptions for addictive drugs preferred (benzodiazepines, opioids, etc.)
	2018	Only electronic prescriptions allowed for addictive drugs

## Data Availability

The data underlying this study's findings are publicly available and can be found at the official websites: Danish Register of Medicinal Product Statistics via the Danish Health Data Authority's site (http://www.medstat.dk), the Norwegian Prescription Database hosted by the Norwegian Institute of Public Health (http://www.norpd.no), the Swedish Prescribed Drug Register available at Socialstyrelsen (http://www.socialstyrelsen.se), and the Nordic Medico-Statistical Committees database (NOMESCO) (http://www.nhwstat.org). Additionally, data is available from the corresponding author upon request.

## Additional Points

*Key points*. (i) The use of antidepressants increased, while the use of anxiolytics decreased in Scandinavia between 2006 and 2021, but there were considerable differences between the three countries studied. (ii) Sweden consistently had the highest prevalence of antidepressants throughout the period, while Denmark saw the largest reduction in anxiolytic use compared to the other two countries. (iii) The prevalence of antidepressant and anxiolytic use was highest in older age groups in Scandinavia, but there was an increasing trend of antidepressant use in younger age groups (5-19 years) in Sweden and Norway. (iv) These differences may reflect variations in healthcare accessibility, national mental health initiatives, and clinical prescribing practices between Scandinavian countries. *Plain Language Summary*. The use of antidepressants and anxiolytics has changed in Scandinavia over recent years; however, there were notable variations between the countries. The aim of this study is to describe and compare the use of antidepressants and anxiolytic drugs in Norway, Sweden, and Denmark from 2006 to 2021, overall, by drug classes and by age groups. Data material included drug prescriptions drawn from prescription registers in each of the three countries. The results showed that the use of antidepressants increased, while the use of anxiolytic drugs decreased in all countries. During the study period, the prevalence of antidepressant use was highest in Sweden. The reduction in anxiolytic use was most marked in Denmark. The use of both drug classes was highest in older age groups (≥65 years). However, there was an increase in antidepressant use among Swedish and Norwegian young people aged 5-19 years, but this was not observed in Denmark. The observed differences in antidepressant and anxiolytic use may reflect differences in healthcare accessibility, national mental health initiatives, and clinical prescribing practices between Scandinavian countries. The findings highlight the importance of investigating the factors underlying the high use of these drugs in older and younger age groups.

## Supplementary Materials

Supplementary Materials Figure S1 shows time trend in the prevalence of antidepressant drug use (number of users per 1000 inhabitants) by age group, drug class, and country. Figure S2 shows time trend in the prevalence of anxiolytic drug use (number of users per 1000 inhabitants) by age group, drug class, and country. Supplementary Table S3 shows the total number of users and prevalence (users per 1000 inhabitants) of “other antidepressants” (ATC N06AX) by drug class and country from 2006 to 2021. Supplementary Table S4 shows time trend in the prevalence (users per 1000 inhabitants) of antidepressant use among women by drug class, age group, and country from 2006 to 2021. Supplementary Table S5 shows time trend in the prevalence (users per 1000 inhabitants) of antidepressant use among women by drug class, age group, and country from 2006 to 2021. Supplementary Table S6 shows time trend in the prevalence (users per 1000 inhabitants) of anxiolytic use among women by drug class, age group, and country from 2006 to 2021. Supplementary Table S7 shows time trend in the prevalence (users per 1000 inhabitants) of anxiolytic use among men by drug class, age group, and country from 2006 to 2021.
