# Supplementary material for: Use of Antidepressant and Anxiolytic Drugs in Scandinavian Countries between 2006 and 2021: A Prescription Database Study
Source: Depress Anxiety. 2024 Jan 5;2024:5448587. doi: 10.1155/2024/5448587 (PMC11919044; doi:10.1155/2024/5448587)
Supplement: Supplementary Materials — Figure S1 shows time trend in the prevalence of antidepressant drug use (number of users per 1000 inhabitants) by age group, drug class, and country. Figure S2 shows time trend in the prevalence of anxiolytic drug use (number of users per 1000 inhabitants) by age group, drug class, and country. Supplementary Table S3 shows the total number of users and prevalence (users per 1000 inhabitants) of “other antidepressants” (ATC N06AX) by drug class and country from 2006 to 2021. Supplementary Table S4 shows time trend in the prevalence (users per 1000 inhabitants) of antidepressant use among women by drug class, age group, and country from 2006 to 2021. Supplementary Table S5 shows time trend in the prevalence (users per 1000 inhabitants) of antidepressant use among women by drug class, age group, and country from 2006 to 2021. Supplementary Table S6 shows time trend in the prevalence (users per 1000 inhabitants) of anxiolytic use among women by drug class, age group, and country from 2006 to 2021. Supplementary Table S7 shows time trend in the prevalence (users per 1000 inhabitants) of anxiolytic use among men by drug class, age group, and country from 2006 to 2021. [file 5448587.f1.zip › Supplementary_material_Table_S3.docx]

**Supplementary Table S3.** Total number of users (Users; No) and prevalence (Prev.; users per 1000 inhabitants) of “other antidepressants” (ATC N06AX) by drug class and country from 2006 to 2021

| Drug group | **2006** | | **2009** | | **2012** | | **2015** | | **2018** | | **2021** | | Relative change  (%) ^a^ | *P* ^b^ |
| --- | --- | --- | --- | --- | --- | --- | --- | --- | --- | --- | --- | --- | --- | --- |
|  | Users | Prev. | Users | Prev. | Users | Prev. | Users | Prev. | Users | Prev. | Users | Prev. |  |  |
| **Other antidepressants (N06AX)** |  |  |  |  |  |  |  |  |  |  |  |  |  |  |
| Norway | 88 876 | 19.1 | 90 568 | 18.8 | 98 709 | 19.7 | 107 668 | 20.7 | 118 284 | 22.3 | 134 022 | 24.9 | + 30.4 |  |
| Sweden | 200 552 | 22.2 | 222 811 | 24.1 | 263 207 | 27.8 | 332 087 | 34.1 | 387 789 | 38.3 | 443 278 | 42.7 | + 92.3 |  |
| Denmark | 128 220 | 23.6 | 150 715 | 27.3 | 174 310 | 31.2 | 166 575 | 29.4 | 173 495 | 30.0 | 191 020 | 32.7 | + 36.4 | ≥ 0.05 |
| **Mianserin (N06AX03)** |  |  |  |  |  |  |  |  |  |  |  |  |  |  |
| Norway | 32 937 | 7.1 | 31289 | 6.5 | 28093 | 5.6 | 25396 | 4.9 | 22022 | 4.2 | 19 280 | 3.6 | - 49.3 |  |
| Sweden | 21 911 | 2.4 | 17 581 | 1.9 | 17 581 | 1.5 | 11 741 | 1.2 | 8 559 | 0.9 | 6 379 | 0.6 | - 75.0 |  |
| Denmark | 22 255 | 4.1 | 23 465 | 4.3 | 21 445 | 3.8 | 15 785 | 2.8 | 10 360 | 1.8 | 8 980 | 1.5 | - 63.4 |  |
| **Mitrazapine (N06AX11)** |  |  |  |  |  |  |  |  |  |  |  |  |  |  |
| Norway | 26 961 | 5.8 | 30394 | 6.3 | 35 704 | 7.1 | 40 849 | 7.9 | 46 259 | 8.7 | 53 529 | 9.9 | + 70.7 |  |
| Sweden | 93 666 | 10.4 | 113 725 | 12.3 | 140 391 | 14.8 | 184 064 | 18.9 | 214 564 | 21.2 | 249 113 | 24.0 | + 130.8 |  |
| Denmark | 58 206 | 10.7 | 70 770 | 12.8 | 80 795 | 14.5 | 79 385 | 14.0 | 83 835 | 14.5 | 87 450 | 15.0 | + 40.2 |  |
| **Bupropion (N06AX12)** |  |  |  |  |  |  |  |  |  |  |  |  |  |  |
| Norway | 6 944 | 1.5 | 5 978 | 1.2 | 10 198 | 2.0 | 12 117 | 2.3 | 11 923 | 2.2 | 12 273 | 2.3 | + 53.3 | ≥ 0.05 |
| Sweden | 20 400 | 2.3 | 15 726 | 1.7 | 20 842 | 2.2 | 32 141 | 3.3 | 40 515 | 4.0 | 52 583 | 5.1 | + 121.7 |  |
| Denmark | 12 620 | 2.3 | 6 105 | 1.1 | 3 925 | 0.7 | 2 305 | 0.4 | 1 845 | 0.3 | 3 185 | 0.6 | - 73.9 |  |
| **Venlafaxine (N06AX16)** |  |  |  |  |  |  |  |  |  |  |  |  |  |  |
| Norway | 27 896 | 6.0 | 28 734 | 6.0 | 30 257 | 6.0 | 32 598 | 6.3 | 32 701 | 6.2 | 33 769 | 6.3 | + 5.0 | ≥ 0.05 |
| Sweden | 57 007 | 6.3 | 58 552 | 6.3 | 71 696 | 7.6 | 85 884 | 8.8 | 90 766 | 9.0 | 89 433 | 8.6 | + 36.5 | ≥ 0.05 |
| Denmark | 37 315 | 6.9 | 43 890 | 8.0 | 58 960 | 10.6 | 54 465 | 9.6 | 48 560 | 8.4 | 45 190 | 7.7 | - 11.6 | ≥ 0.05 |

| Drug group | **2006** | | **2009** | | **2012** | | **2015** | | **2018** | | **2021** | | Relative change  (%) ^a^ | *P* ^b^ |
| --- | --- | --- | --- | --- | --- | --- | --- | --- | --- | --- | --- | --- | --- | --- |
|  | Users | Prev. | Users | Prev. | Users | Prev. | Users | Prev. | Users | Prev. | Users | Prev. |  |  |
| **Duloxetine (N06AX21)** |  |  |  |  |  |  |  |  |  |  |  |  |  |  |
| Norway | 1 590 | 0.3 | 2 419 | 0.5 | 3 201 | 0.6 | 4 660 | 0.9 | 7 201 | 1.4 | 8 602 | 1.6 | + 433.3 |  |
| Sweden | 21 836 | 2.4 | 35 352 | 3.8 | 36 508 | 3.9 | 44 425 | 4.6 | 54 346 | 5.4 | 64 343 | 6.2 | + 158.3 |  |
| Denmark | 11 440 | 2.1 | 20 500 | 3.7 | 21 040 | 3.8 | 22 530 | 4.0 | 34 375 | 6.0 | 48 375 | 8.3 | + 295.2 |  |

**Notes**: Prevalence less than 2 users per 1000 inhabitants are not presented in the table. The total count of N06AX differs from the subgroup totals because the numbers are weighted and presented per 1000 inhabitants. The table display data points every three years for ease of presentation, but the statistical trend analysis and graphs are based on data from each year.

*Continuation* **Table S3**

^a^ Percentage differences in year 2021 compared to year 2006; differences ≤ 1% are omitted from the table

^b^ Jonckheere-Terpstra trend test p-value for trend analysis ≥ 0.05
