# Supplementary material for: Use of Antidepressant and Anxiolytic Drugs in Scandinavian Countries between 2006 and 2021: A Prescription Database Study
Source: Depress Anxiety. 2024 Jan 5;2024:5448587. doi: 10.1155/2024/5448587 (PMC11919044; doi:10.1155/2024/5448587)
Supplement: Supplementary Materials — Figure S1 shows time trend in the prevalence of antidepressant drug use (number of users per 1000 inhabitants) by age group, drug class, and country. Figure S2 shows time trend in the prevalence of anxiolytic drug use (number of users per 1000 inhabitants) by age group, drug class, and country. Supplementary Table S3 shows the total number of users and prevalence (users per 1000 inhabitants) of “other antidepressants” (ATC N06AX) by drug class and country from 2006 to 2021. Supplementary Table S4 shows time trend in the prevalence (users per 1000 inhabitants) of antidepressant use among women by drug class, age group, and country from 2006 to 2021. Supplementary Table S5 shows time trend in the prevalence (users per 1000 inhabitants) of antidepressant use among women by drug class, age group, and country from 2006 to 2021. Supplementary Table S6 shows time trend in the prevalence (users per 1000 inhabitants) of anxiolytic use among women by drug class, age group, and country from 2006 to 2021. Supplementary Table S7 shows time trend in the prevalence (users per 1000 inhabitants) of anxiolytic use among men by drug class, age group, and country from 2006 to 2021. [file 5448587.f1.zip › Supplementary_material_Table_S6-S7.docx]

**Supplementary Table S6.** Time trend in the prevalence (Prev.; users per 1000 inhabitants) of anxiolytic use among women by drug class, age groups, and country from 2006 to 2021.

|  | **Drug class (ATC)** | **Age**  **(years)** | **2006** | **2009** | **2012** | **2015** | **2018** | **2021** | **Relative change (%)^a^** | ***P* ^b^** |
| --- | --- | --- | --- | --- | --- | --- | --- | --- | --- | --- |
| **Sweden** |  |  |  |  |  |  |  |  |  |  |
|  | N05B | 15-19 | 14.4 | 18.0 | 23.1 | 34.4 | 38.5 | 38.9 | + 170.0 |  |
|  |  |  |  |  |  |  |  |  |  |  |
|  |  | 20-44 | 41.0 | 45.0 | 51.3 | 61.0 | 58.4 | 51.7 | + 26.1 |  |
|  |  |  |  |  |  |  |  |  |  |  |
|  |  | 45-64 | 78.4 | 78.5 | 81.0 | 85.9 | 79.4 | 70.5 | - 10.1 | ≥ 0.05 |
|  |  |  |  |  |  |  |  |  |  |  |
|  |  | 65-74 | 116.7 | 115.7 | 112.9 | 106.9 | 93.9 | 81.6 | - 30.0 |  |
|  |  |  |  |  |  |  |  |  |  |  |
|  |  | ≥ 75 | 213.2 | 205.7 | 202.1 | 193.9 | 177.0 | 158.2 | - 25.8 |  |
|  |  |  |  |  |  |  |  |  |  |  |
|  | N05BA | 15-19 | 4.3 | 4.5 | 4.3 | 4.4 | 3.6 | 2.5 | - 41.9 | ≥ 0.05 |
|  |  |  |  |  |  |  |  |  |  |  |
|  |  | 20-44 | 24.2 | 24.1 | 25.3 | 26.3 | 22.6 | 17.1 | - 29.3 | ≥ 0.05 |
|  |  |  |  |  |  |  |  |  |  |  |
|  |  | 45-64 | 78.7 | 72.5 | 69.7 | 65.6 | 56.2 | 44.4 | - 43.6 |  |
|  |  |  |  |  |  |  |  |  |  |  |
|  |  | 65-74 | 98.6 | 92.4 | 87.2 | 79.2 | 67.3 | 55.5 | - 43.7 |  |
|  |  |  |  |  |  |  |  |  |  |  |
|  |  | ≥ 75 | 188.8 | 179.4 | 176.2 | 170.8 | 157.2 | 141.6 | - 25.0 |  |
|  |  |  |  |  |  |  |  |  |  |  |
|  | N05BB | 15-19 | 10.4 | 14.4 | 19.6 | 31.0 | 35.4 | 36.6 | + 251.9 |  |
|  |  |  |  |  |  |  |  |  |  |  |
|  |  | 20-44 | 19.8 | 25.0 | 30.7 | 39.7 | 39.3 | 36.2 | + 82.8 | ≥ 0.05 |
|  |  |  |  |  |  |  |  |  |  |  |
|  |  | 45-64 | 24.1 | 30.2 | 35.5 | 43.7 | 42.6 | 41.0 | + 70.1 |  |
|  |  |  |  |  |  |  |  |  |  |  |
|  |  | 65-74 | 24.0 | 30.5 | 33.2 | 35.0 | 32.1 | 30.2 | + 25.8 | ≥ 0.05 |
|  |  |  |  |  |  |  |  |  |  |  |
|  |  | ≥ 75 | 11.5 | 12.5 | 12.1 | 11.0 | 8.6 | 6.9 | - 40.0 | ≥ 0.05 |
|  |  |  |  |  |  |  |  |  |  |  |
|  | N05BE | 15-19 | 0.3 | 0.2 | 0.1 | 0.2 | 0.4 | 0.5 | + 66.7 | ≥ 0.05 |
|  |  |  |  |  |  |  |  |  |  |  |
|  |  | 20-44 | 1.2 | 0.8 | 0.7 | 0.9 | 1.3 | 1.9 | + 58.3 | ≥ 0.05 |
|  |  |  |  |  |  |  |  |  |  |  |
|  |  | 45-64 | 1.9 | 1.4 | 1.1 | 1.1 | 1.2 | 1.4 | - 26.3 | ≥ 0.05 |
|  |  |  |  |  |  |  |  |  |  |  |
|  |  | 65-74 | 1.4 | 1.1 | 0.9 | 0.9 | 0.9 | 1.0 | - 42.9 | ≥ 0.05 |
|  |  |  |  |  |  |  |  |  |  |  |
|  |  | ≥ 75 | 1.5 | 1.0 | 0.8 | 0.9 | 1.1 | 1.4 | - | - |
| **Norway** |  |  |  |  |  |  |  |  |  |  |
|  | N05B | 15-19 | 8.9 | 8.8 | 8.4 | 8.2 | 8.0 | 8.2 | - 7.9 |  |
|  |  |  |  |  |  |  |  |  |  |  |
|  |  | 20-44 | 44.4 | 44.0 | 41.2 | 39.3 | 38.0 | 35.9 | - 19.1 |  |
|  |  |  |  |  |  |  |  |  |  |  |
|  |  | 45-64 | 116.3 | 110.3 | 101.6 | 91.0 | 79.4 | 72.6 | - 37.6 |  |
|  |  |  |  |  |  |  |  |  |  |  |
|  |  | 65-74 | 178.0 | 163.7 | 150.1 | 137.5 | 125.4 | 117.2 | + 34.2 |  |
|  |  |  |  |  |  |  |  |  |  |  |
|  |  | ≥ 75 | 199.8 | 194.8 | 189.7 | 176.0 | 159.5 | 150.2 | - 24.8 |  |
|  |  |  |  |  |  |  |  |  |  |  |
|  | N05BA | 15-19 | 5.9 | 5.8 | 4.9 | 4.4 | 4.4 | 4.1 | - 30.5 |  |
|  |  |  |  |  |  |  |  |  |  |  |
|  |  | 20-44 | 39.3 | 38.7 | 35.3 | 32.9 | 32.4 | 29.6 | - 24.7 |  |
|  |  |  |  |  |  |  |  |  |  |  |
|  |  | 45-64 | 109.9 | 103.7 | 93.9 | 83.0 | 72.4 | 65.2 | - 40.7 |  |
|  |  |  |  |  |  |  |  |  |  |  |
|  |  | 65-74 | 170.7 | 156.1 | 142.0 | 129.2 | 117.8 | 109.4 | - 35.9 |  |
|  |  |  |  |  |  |  |  |  |  |  |
|  |  | ≥ 75 | 190.3 | 185.4 | 179.4 | 166.0 | 151.7 | 142.7 | - 25.0 |  |
|  |  |  |  |  |  |  |  |  |  |  |
|  | N05BB | 15-19 | 3.0 | 3.2 | 3.6 | 4.0 | 3.7 | 4.2 | + 40.0 |  |
|  |  |  |  |  |  |  |  |  |  |  |
|  |  | 20-44 | 6.5 | 6.9 | 7.5 | 8.0 | 6.8 | 7.6 | + 16.9 | ≥ 0.05 |
|  |  |  |  |  |  |  |  |  |  |  |
|  |  | 45-64 | 9.1 | 9.7 | 11.0 | 11.2 | 9.2 | 9.7 | + 6.6 | ≥ 0.05 |
|  |  |  |  |  |  |  |  |  |  |  |
|  |  | 65-74 | 11.0 | 11.3 | 12.3 | 12.0 | 10.2 | 10.6 | - 3.6 | ≥ 0.05 |
|  |  |  |  |  |  |  |  |  |  |  |
|  |  | ≥ 75 | 14.0 | 13.5 | 14.9 | 14.0 | 10.3 | 10.2 | - 27.1 | ≥ 0.05 |
|  |  |  |  |  |  |  |  |  |  |  |
|  | N05BE | 15-19 | 0.1 | 0.1 | 0.1 | 0.1 | 0.1 | 0.1 | - | - |
|  |  |  |  |  |  |  |  |  |  |  |
|  |  | 20-44 | 0.7 | 0.6 | 0.6 | 0.5 | 0.4 | 0.5 | - 28.6 |  |
|  |  |  |  |  |  |  |  |  |  |  |
|  |  | 45-64 | 1.4 | 0.9 | 1.0 | 0.8 | 0.6 | 0.6 | - 57.1 |  |
|  |  |  |  |  |  |  |  |  |  |  |
|  |  | 65-74 | 1.1 | 0.9 | 1.0 | 1.0 | 0.9 | 0.8 | - 27.3 | ≥ 0.05 |
|  |  |  |  |  |  |  |  |  |  |  |
|  |  | ≥ 75 | 0.8 | 0.7 | 0.8 | 0.6 | 0.6 | 0.7 | - 12.5 | ≥ 0.05 |
| **Denmark** |  |  |  |  |  |  |  |  |  |  |
|  | N05B | 15-19 | 7.3 | 5.7 | 4.8 | 4.7 | 4.0 | 4.5 | - 38.4 |  |
|  |  |  |  |  |  |  |  |  |  |  |
|  |  | 20-44 | 29.1 | 22.1 | 18.6 | 16.0 | 13.9 | 12.6 | - 56.7 |  |
|  |  |  |  |  |  |  |  |  |  |  |
|  |  | 45-64 | 94.5 | 71.2 | 55.9 | 45.0 | 34.8 | 27.0 | - 71.4 |  |
|  |  |  |  |  |  |  |  |  |  |  |
|  |  | 65-74 | 158.6 | 120.8 | 92.3 | 71.3 | 56.8 | 46.2 | - 70.9 |  |
|  |  |  |  |  |  |  |  |  |  |  |
|  |  | ≥ 75 | 189.1 | 158.9 | 132.7 | 105.6 | 82.6 | 66.3 | - 64.9 |  |
|  |  |  |  |  |  |  |  |  |  |  |
|  | N05BA | 15-19 | 6.7 | 5.1 | 4.1 | 4.1 | 3.4 | 3.6 | - 46.3 |  |
|  |  |  |  |  |  |  |  |  |  |  |
|  |  | 20-44 | 28.2 | 21.3 | 17.6 | 14.7 | 12.5 | 10.8 | -61.7 |  |
|  |  |  |  |  |  |  |  |  |  |  |
|  |  | 45-64 | 93.0 | 69.8 | 54.3 | 43.2 | 32.9 | 24.9 | - 73.2 |  |
|  |  |  |  |  |  |  |  |  |  |  |
|  |  | 65-74 | 156.3 | 118.8 | 90.1 | 69.0 | 54.5 | 43.7 | - 72.0 |  |
|  |  |  |  |  |  |  |  |  |  |  |
|  |  | ≥ 75 | 184.7 | 154.6 | 128.2 | 101.7 | 78.6 | 62.6 | - 66.1 |  |
|  |  |  |  |  |  |  |  |  |  |  |
|  | N05BB | 15-19 | 0.5 | 0.4 | 0.5 | 0.6 | 0.5 | 0.9 | + 80.0 | ≥ 0.05 |
|  |  |  |  |  |  |  |  |  |  |  |
|  |  | 20-44 | 0.9 | 0.8 | 1.0 | 1.4 | 1.5 | 1.9 | + 111.1 |  |
|  |  |  |  |  |  |  |  |  |  |  |
|  |  | 45-64 | 1.8 | 1.5 | 1.8 | 2.1 | 2.1 | 2.3 | + 27.3 |  |
|  |  |  |  |  |  |  |  |  |  |  |
|  |  | 65-74 | 2.9 | 2.4 | 2.7 | 2.6 | 2.6 | 2.7 | - 6.9 | ≥ 0.05 |
|  |  |  |  |  |  |  |  |  |  |  |
|  |  | ≥ 75 | 5.8 | 5.3 | 5.5 | 4.4 | 4.5 | 3.9 | - 32.8 |  |
|  |  |  |  |  |  |  |  |  |  |  |
|  | N05BE | 15-19 | 0.2 | 0.2 | 0.2 | 0.0 | 0.1 | 0.0 | - 100.0 | ≥ 0.05 |
|  |  |  |  |  |  |  |  |  |  |  |
|  |  | 20-44 | 0.3 | 0.2 | 0.2 | 0.2 | 0.2 | 0.2 | - 33.3 | ≥ 0.05 |
|  |  |  |  |  |  |  |  |  |  |  |
|  |  | 45-64 | 0.5 | 0.5 | 0.3 | 0.3 | 0.3 | 0.3 | - 40.0 | ≥ 0.05 |
|  |  |  |  |  |  |  |  |  |  |  |
|  |  | 65-74 | 0.4 | 0.5 | 0.3 | 0.3 | 0.3 | 0.3 | - 25.0 | ≥ 0.05 |
|  |  |  |  |  |  |  |  |  |  |  |
|  |  | ≥ 75 | 0.2 | 0.3 | 0.2 | 0.1 | 0.1 | 0.1 | - 50.0 | ≥ 0.05 |

**Abbreviations:** BZDs: Benzodiazepine derivatives; Diphenylmet. derivatives: Diphenylmethane derivatives; Azasp. derviates: Azaspirodecanedione derivates

The table displays data points every three years for ease of presentation, but the statistical trend analysis and graphs are based on data from each year.

The table displays data points every three years for ease of presentation, but the statistical trend analysis and graphs are based on data from each year.

^a^ Percentage differences in year 2021 compared to year 2006; differences ≤ 1% are omitted from the table

^b^ Jonckheere-Terpstra trend test p-value for trend analysis ≥ 0.05

**Supplementary Table S7.** Time trend in the prevalence (Prev.; users per 1000 inhabitants) of anxiolytic use among men by drug class, age groups, and country from 2006 to 2021.

|  | **Drug class (ATC)** | **Age**  **(years)** | **2006** | **2009** | **2012** | **2015** | **2018** | **2021** | **Relative change (%)^a^** | ***P* ^b^** |
| --- | --- | --- | --- | --- | --- | --- | --- | --- | --- | --- |
| **Sweden** |  |  |  |  |  |  |  |  |  |  |
|  | N05B | 15-19 | 7.9 | 9.7 | 12.7 | 15.8 | 16.5 | 14.4 | + 82.3 | ≥ 0.05 |
|  |  |  |  |  |  |  |  |  |  |  |
|  |  | 20-44 | 24.7 | 27.5 | 30.3 | 34.9 | 31.7 | 27.3 | + 10.5 | ≥ 0.05 |
|  |  |  |  |  |  |  |  |  |  |  |
|  |  | 45-64 | 47.5 | 48.8 | 49.7 | 50.4 | 44.7 | 38.3 | - 19.4 | ≥ 0.05 |
|  |  |  |  |  |  |  |  |  |  |  |
|  |  | 65-74 | 69.3 | 68.7 | 66.4 | 63.6 | 56.6 | 49.3 | - 28.9 |  |
|  |  |  |  |  |  |  |  |  |  |  |
|  |  | ≥ 75 | 142.1 | 137.3 | 135.0 | 128.9 | 115.5 | 99.2 | - 30.2 |  |
|  |  |  |  |  |  |  |  |  |  |  |
|  | N05BA | 15-19 | 3.0 | 3.3 | 3.4 | 2.8 | 2.2 | 1.6 | - 46.7 | ≥ 0.05 |
|  |  |  |  |  |  |  |  |  |  |  |
|  |  | 20-44 | 15.4 | 15.7 | 16.3 | 16.3 | 13.2 | 9.6 | - 37.7 | ≥ 0.05 |
|  |  |  |  |  |  |  |  |  |  |  |
|  |  | 45-64 | 35.9 | 34.8 | 33.9 | 31.7 | 26.6 | 20.9 | - 41.8 |  |
|  |  |  |  |  |  |  |  |  |  |  |
|  |  | 65-74 | 56.7 | 53.9 | 50.7 | 47.5 | 41.9 | 35.3 | - 37.7 |  |
|  |  |  |  |  |  |  |  |  |  |  |
|  |  | ≥ 75 | 118.5 | 112.8 | 112.0 | 109.2 | 99.9 | 87.3 | - 26.3 |  |
|  |  |  |  |  |  |  |  |  |  |  |
|  | N05BB | 15-19 | 4.1 | 6.8 | 9.7 | 13.5 | 14.7 | 12.9 | + 24.6 |  |
|  |  |  |  |  |  |  |  |  |  |  |
|  |  | 20-44 | 10.7 | 13.8 | 16.2 | 20.9 | 19.9 | 18.2 | + 70.1 | ≥ 0.05 |
|  |  |  |  |  |  |  |  |  |  |  |
|  |  | 45-64 | 13.9 | 17.2 | 19.2 | 22.0 | 20.4 | 18.8 | + 35.3 | ≥ 0.05 |
|  |  |  |  |  |  |  |  |  |  |  |
|  |  | 65-74 | 15.3 | 18.4 | 19.6 | 19.6 | 17.4 | 15.7 | + 2.6 | ≥ 0.05 |
|  |  |  |  |  |  |  |  |  |  |  |
|  |  | ≥ 75 | 30.4 | 31.6 | 29.9 | 25.4 | 19.2 | 14.4 | - 52.6 | ≥ 0.05 |
|  |  |  |  |  |  |  |  |  |  |  |
|  | N05BE | 15-19 | 0.1 | 0.1 | 0.1 | 0.1 | 0.1 | 0.1 | - | - |
|  |  |  |  |  |  |  |  |  |  |  |
|  |  | 20-44 | 1.0 | 0.7 | 0.6 | 0.9 | 1.0 | 1.2 | + 20.0 | ≥ 0.05 |
|  |  |  |  |  |  |  |  |  |  |  |
|  |  | 45-64 | 1.6 | 1.2 | 0.9 | 1.0 | 1.0 | 1.1 | - 31.3 | ≥ 0.05 |
|  |  |  |  |  |  |  |  |  |  |  |
|  |  | 65-74 | 1.2 | 0.9 | 0.7 | 0.7 | 0.8 | 0.9 | - 25.0 | ≥ 0.05 |
|  |  |  |  |  |  |  |  |  |  |  |
|  |  | ≥ 75 | 1.0 | 0.7 | 0.6 | 0.5 | 0.5 | 0.5 | - 50.0 | ≥ 0.05 |
| **Norway** |  |  |  |  |  |  |  |  |  |  |
|  | N05B | 15-19 | 5.0 | 5.7 | 5.3 | 4.9 | 4.8 | 4.8 | - 36.0 | ≥ 0.05 |
|  |  |  |  |  |  |  |  |  |  |  |
|  |  | 20-44 | 30.2 | 31.5 | 28.5 | 26.4 | 25.3 | 24.6 | - 18.5 |  |
|  |  |  |  |  |  |  |  |  |  |  |
|  |  | 45-64 | 63.7 | 62.9 | 58.0 | 52.9 | 46.4 | 43.8 | - 31.2 |  |
|  |  |  |  |  |  |  |  |  |  |  |
|  |  | 65-74 | 89.5 | 82.2 | 76.4 | 71.5 | 65.1 | 62.2 | - 30.5 |  |
|  |  |  |  |  |  |  |  |  |  |  |
|  |  | ≥ 75 | 121.0 | 118.3 | 110.1 | 99.6 | 87.5 | 81.9 | - 32.2 |  |
|  |  |  |  |  |  |  |  |  |  |  |
|  | N05BA | 15-19 | 3.5 | 4.0 | 3.5 | 3.0 | 3.0 | 2.5 | - 28.6 |  |
|  |  |  |  |  |  |  |  |  |  |  |
|  |  | 20-44 | 26.9 | 28.1 | 24.7 | 25.7 | 21.7 | 20.4 | - 24.2 |  |
|  |  |  |  |  |  |  |  |  |  |  |
|  |  | 45-64 | 59.7 | 58.8 | 53.4 | 48.2 | 42.3 | 39.4 | - 34.0 |  |
|  |  |  |  |  |  |  |  |  |  |  |
|  |  | 65-74 | 83.8 | 76.5 | 70.3 | 65.7 | 60.0 | 56.6 | - 32.6 |  |
|  |  |  |  |  |  |  |  |  |  |  |
|  |  | ≥ 75 | 109.0 | 105.7 | 97.3 | 87.1 | 77.8 | 73.5 | - 32.6 |  |
|  |  |  |  |  |  |  |  |  |  |  |
|  | N05BB | 15-19 | 1.5 | 1.7 | 1.9 | 2.0 | 1.9 | 2.4 | - 60.0 |  |
|  |  |  |  |  |  |  |  |  |  |  |
|  |  | 20-44 | 4.1 | 4.5 | 4.7 | 4.9 | 4.3 | 4.9 | + 19.5 | ≥ 0.05 |
|  |  |  |  |  |  |  |  |  |  |  |
|  |  | 45-64 | 5.1 | 5.6 | 6.1 | 6.2 | 5.0 | 5.4 | + 5.9 | ≥ 0.05 |
|  |  |  |  |  |  |  |  |  |  |  |
|  |  | 65-74 | 7.1 | 7.1 | 7.9 | 7.3 | 6.3 | 6.6 | - 7.0 | ≥ 0.05 |
|  |  |  |  |  |  |  |  |  |  |  |
|  |  | ≥ 75 | 14.9 | 15.6 | 15.7 | 14.9 | 11.3 | 9.9 | - 33.6 | ≥ 0.05 |
|  |  |  |  |  |  |  |  |  |  |  |
|  | N05BE | 15-19 | 0.1 | 0.1 | 0.0 | 0.1 | 0.1 | 0.0 | - | - |
|  |  |  |  |  |  |  |  |  |  |  |
|  |  | 20-44 | 0.6 | 0.5 | 0.5 | 0.4 | 0.4 | 0.4 | - 33.3 |  |
|  |  |  |  |  |  |  |  |  |  |  |
|  |  | 45-64 | 0.9 | 0.7 | 0.6 | 0.5 | 0.4 | 0.4 | - 55.6 |  |
|  |  |  |  |  |  |  |  |  |  |  |
|  |  | 65-74 | 0.6 | 0.5 | 0.6 | 0.6 | 0.5 | 0.5 | - 16.7 | ≥ 0.05 |
|  |  |  |  |  |  |  |  |  |  |  |
|  |  | ≥ 75 | 0.3 | 0.3 | 0.4 | 0.4 | 0.4 | 0.3 | - | ≥ 0.05 |
| **Denmark** |  |  |  |  |  |  |  |  |  |  |
|  | N05B | 15-19 | 4.2 | 3.9 | 3.2 | 2.8 | 2.7 | 2.7 | - 35.7 |  |
|  |  |  |  |  |  |  |  |  |  |  |
|  |  | 20-44 | 19.6 | 15.3 | 13.0 | 10.9 | 9.1 | 8.2 | - 58.2 |  |
|  |  |  |  |  |  |  |  |  |  |  |
|  |  | 45-64 | 53.9 | 42.7 | 34.8 | 28.0 | 21.8 | 16.9 | - 68.6 |  |
|  |  |  |  |  |  |  |  |  |  |  |
|  |  | 65-74 | 78.1 | 59.1 | 46.3 | 37.9 | 31.3 | 25.6 | - 67.2 |  |
|  |  |  |  |  |  |  |  |  |  |  |
|  |  | ≥ 75 | 119.1 | 96.8 | 81.7 | 61.8 | 46.0 | 36.8 | - 69.1 |  |
|  |  |  |  |  |  |  |  |  |  |  |
|  | N05BA | 15-19 | 3.9 | 3.4 | 2.8 | 2.4 | 2.4 | 2.2 | - 43.6 |  |
|  |  |  |  |  |  |  |  |  |  |  |
|  |  | 20-44 | 19.0 | 14.7 | 12.2 | 10.1 | 8.3 | 7.0 | - 63.2 |  |
|  |  |  |  |  |  |  |  |  |  |  |
|  |  | 45-64 | 52.9 | 41.8 | 33.6 | 26.8 | 20.5 | 15.7 | - 70.3 |  |
|  |  |  |  |  |  |  |  |  |  |  |
|  |  | 65-74 | 76.4 | 57.6 | 44.3 | 36.1 | 29.8 | 24.1 | - 68.4 |  |
|  |  |  |  |  |  |  |  |  |  |  |
|  |  | ≥ 75 | 113.8 | 91.8 | 76.5 | 57.4 | 42.2 | 33.6 | - 70.5 |  |
|  |  |  |  |  |  |  |  |  |  |  |
|  | N05BB | 15-19 | 0.3 | 0.4 | 0.3 | 0.4 | 0.3 | 0.5 | + 66.7 | ≥ 0.05 |
|  |  |  |  |  |  |  |  |  |  |  |
|  |  | 20-44 | 0.6 | 0.6 | 0.7 | 0.9 | 0.9 | 1.2 | + 100.0 |  |
|  |  |  |  |  |  |  |  |  |  |  |
|  |  | 45-64 | 1.1 | 1.0 | 1.2 | 1.2 | 1.4 | 1.3 | + 18.2 | ≥ 0.05 |
|  |  |  |  |  |  |  |  |  |  |  |
|  |  | 65-74 | 2.0 | 1.6 | 2.2 | 1.9 | 1.7 | 1.6 | - 20.0 | ≥ 0.05 |
|  |  |  |  |  |  |  |  |  |  |  |
|  |  | ≥ 75 | 6.4 | 5.4 | 5.4 | 4.9 | 4.1 | 3.4 | - 46.9 |  |
|  |  |  |  |  |  |  |  |  |  |  |
|  | N05BE | 15-19 | 0.0 | 0.0 | 0.0 | 0.0 | 0.0 | 0.0 | - |  |
|  |  |  |  |  |  |  |  |  |  |  |
|  |  | 20-44 | 0.2 | 0.2 | 0.2 | 0.1 | 0.1 | 0.1 | - 50.0 | ≥ 0.05 |
|  |  |  |  |  |  |  |  |  |  |  |
|  |  | 45-64 | 0.4 | 0.3 | 0.3 | 0.2 | 0.2 | 0.2 | - 50.0 |  |
|  |  |  |  |  |  |  |  |  |  |  |
|  |  | 65-74 | 0.1 | 0.3 | 0.2 | 0.2 | 0.1 | 0.1 | - | - |
|  |  |  |  |  |  |  |  |  |  |  |
|  |  | ≥ 75 | 0.1 | 0.1 | 0.0 | 0.0 | 0.1 | 0.0 | - | - |

**Abbreviations:** BZDs: Benzodiazepine derivatives; Diphenylmet. derivatives: Diphenylmethane derivatives; Azasp. derviates: Azaspirodecanedione derivates

The table displays data points every three years for ease of presentation, but the statistical trend analysis and graphs are based on data from each year.

^a^ Percentage differences in year 2021 compared to year 2006; differences ≤ 1% are omitted from the table

^b^ Jonckheere-Terpstra trend test p-value for trend analysis ≥ 0.05
